# Supplementary material for: Contractile injection systems facilitate sporogenic differentiation of Streptomyces davawensis through the action of a phage tapemeasure protein-related effector
Source: Nat Commun. 2024 May 24;15:4442. doi: 10.1038/s41467-024-48834-9 (PMC11126660; doi:10.1038/s41467-024-48834-9)
Supplement: Supplementary file 1 — Supplementary Information [file 41467_2024_48834_MOESM1_ESM.pdf]

Supplementary Information

Contractile injection systems facilitate sporogenic differentiation of *Streptomyces davawensis* through the action of a phage tapemeasure protein-related effector.

Nagakubo, T. *et al.*

Contents:

Supplementary Figures 1-19
Supplementary Tables 1-4
Supplementary Notes
Supplementary References

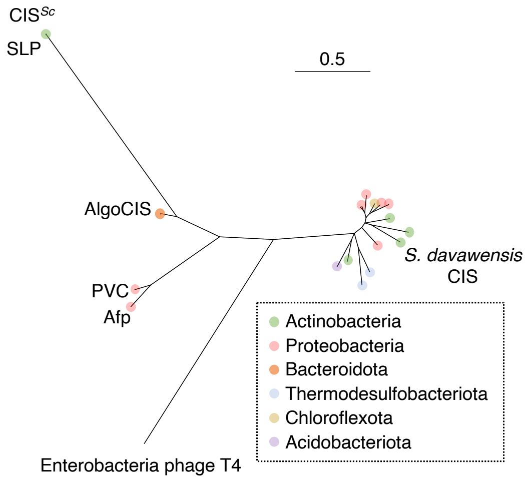

Supplementary Figure 1. A phylogenetic tree for CIS tube proteins.

A phylogenetic tree for CIS tube proteins was constructed by the same method with Fig. 1b. Proteins used in this analysis are listed in Supplementary Table 3.

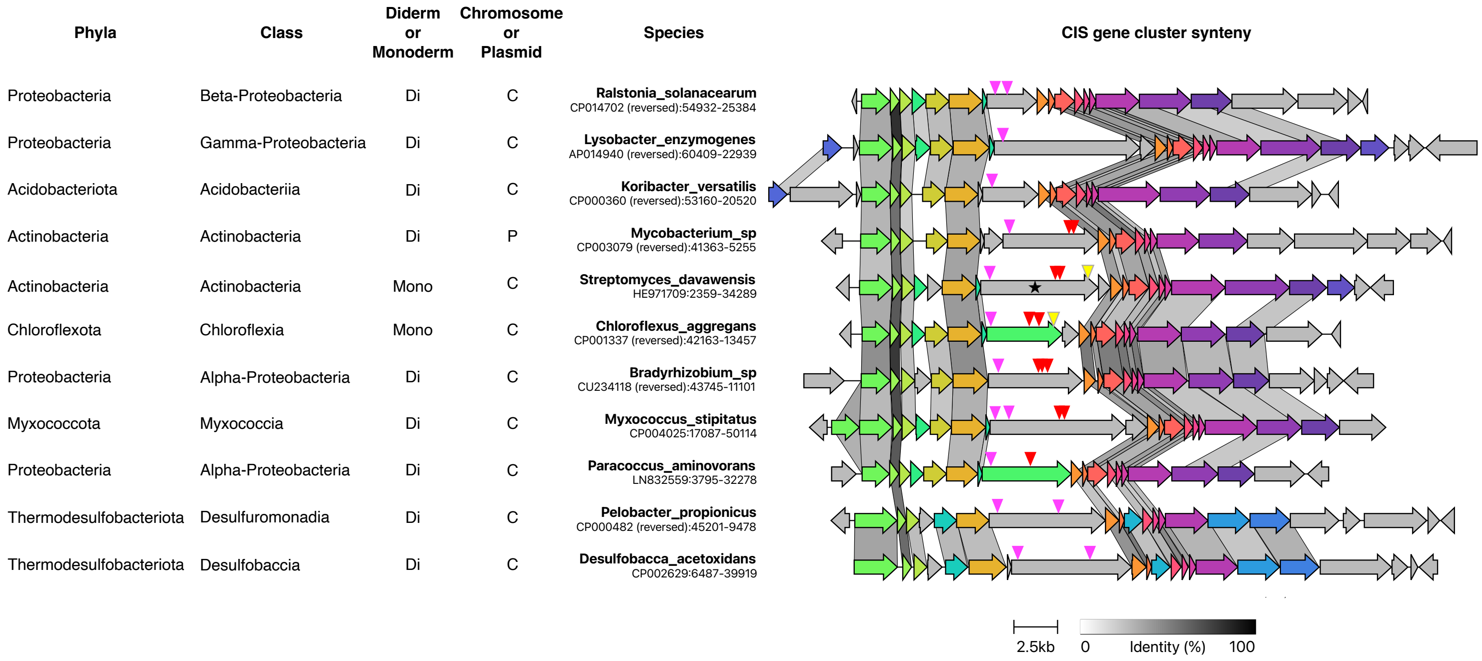

Supplementary Figure 2. CIS gene clusters related to *S. davawensis* CIS.

Structures of the CIS-related gene clusters closely related to *S. davawensis* CISs were compared. Synteny analysis was performed by clinker[1]. Coloured arrows indicate genes sharing amino acid sequence homology with each other. An arrow with black star indicates *BN159_7576* (*tme*) of *S. davawensis*. Magenta, red, and yellow arrowheads indicate the positions of DUF4157 domain, predicted transmembrane helices, and nuclease domain, respectively, encoded in the *tme*-like genes. Transmembrane helices were predicted by TMHMM-2.0[2].

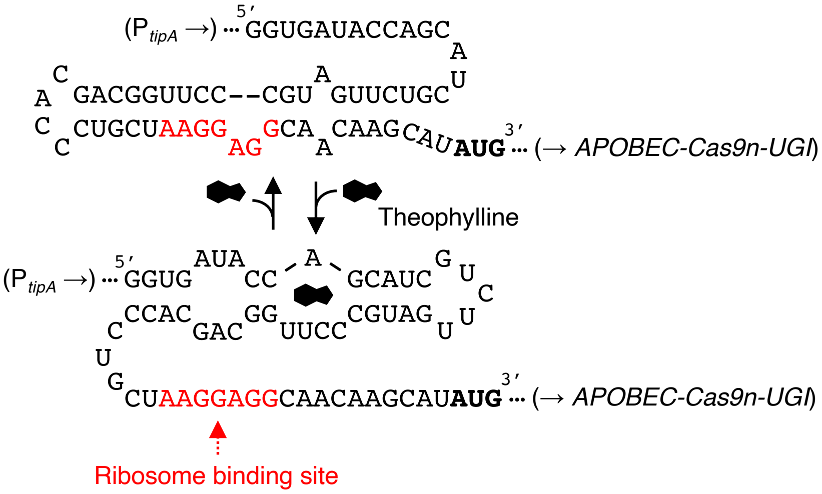

Supplementary Figure 3. Scheme of theophylline-inducible expression of APOBEC-Cas9n-UGI enzyme.

A synthetic riboswitch was introduced into the CRISPR-based genome editing system for gene knockout in *S. davawensis*. Theophylline alters the secondary structure of the stem-loop region that interfere with ribosome binding to the mRNA, thereby allowing for translation initiation.

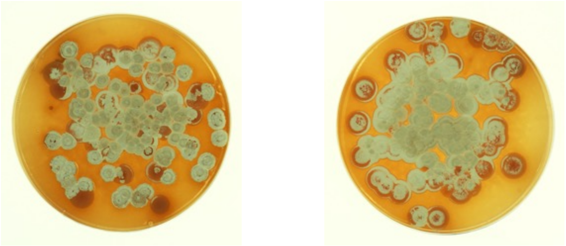

Supplementary Figure 4. CIS loss does not affect spore formation of *S. davawensis* in low density culture.

Low density cultures of the *S. davawensis* strains are shown. These are representative images for three independent cultures of each strain. Approximately 100 spores of each strain were spread onto MS medium and incubated at 30°C for 5 days. Aerial mycelia erection and spore formation are indicated by white and gray regions, respectively, on the colonies.

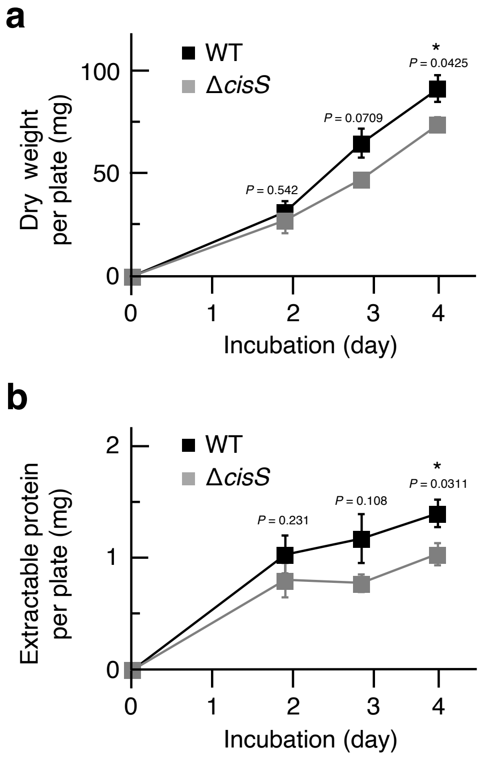

Supplementary Figure 5. Additional biomass measurement of the *S. davawensis* cultures.

Biomass measurement in Fig. 2a was validated by measuring (a) dry weight and (b) amounts of extractable and soluble proteins of *S. davawensis* colonies. Colonies were scraped off a cellophane membrane placed on a solid MS medium and then subjected to either drying or sonication in 10 mM HEPES-NaOH (pH7.4) buffer. To quantify the amounts of extractable and soluble proteins, the lysates were centrifuged at 7,000 × *g* and then the supernatant was analysed by the Bradford method. Values indicate the means ± S.D. for three independent cultures. *P* values were calculated by two-sided *t*-test with Welch's correction. *, time points at which *P* values reached a significance threshold 0.05. Source data are provided as a Source Data file.

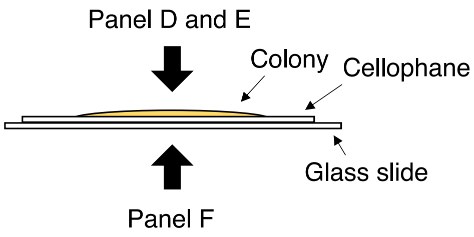

Supplementary Figure 6. Observation condition for Figure 2.

Scheme of microscopic analyses in Fig. 2d-f is shown. Colonies grown on a cellophane placed onto MS medium was peeled off and then placed onto a glass slide. Arrows indicate the directions in which the colonies were observed using microscopes.

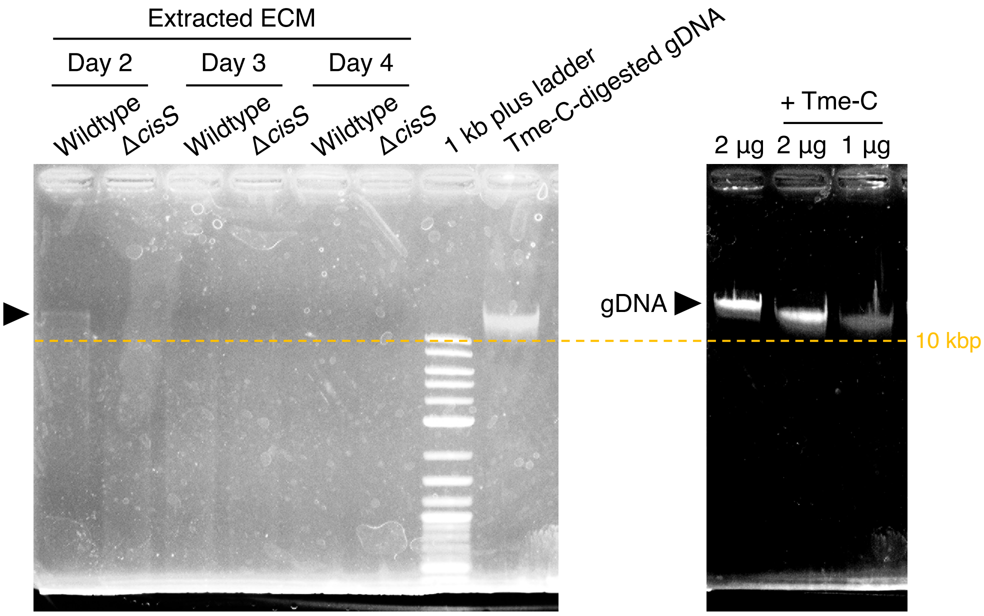

Supplementary Figure 7. Detection of high molecular weight eDNA in the extracted extracellular matrix of *S. davawensis* colony.

All samples in this figure were migrated in the same gel and two panels are displayed in different pixel intensities. (Left) eDNA in the ECM extracts were concentrated (10-fold) by ethanol precipitation and subjected to agarose gel electrophoresis. High molecular weight eDNA (>10 kbp) was detected in the wildtype ECM extract on day 2. An arrowhead indicates the band of high molecular weight eDNA. (Right) The extracted genomic DNA (gDNA) was incubated with the isolated Tme-C at 30°C for 3 h. gDNA concentration in the reaction mixture was 50 or 100 ng/µL, and 20 µL of the mixture was applied to each lane. An arrowhead indicates the band of gDNA. All samples were mixed with SDS (0.05% (w/v)) in prior to the electrophoresis.

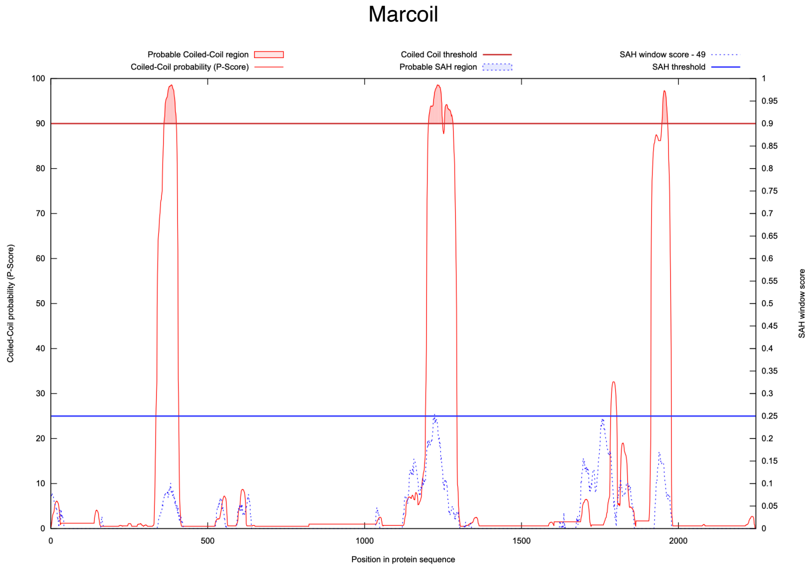
Marcoil
Probable Coiled-Coil region
Coiled-Coil probability (P-Score)
Coiled Coil threshold
Probable SAH region
SAH window score - 49
SAH threshold
Coiled-Coil probability (P-Score)
SAH window score
Position in protein sequence

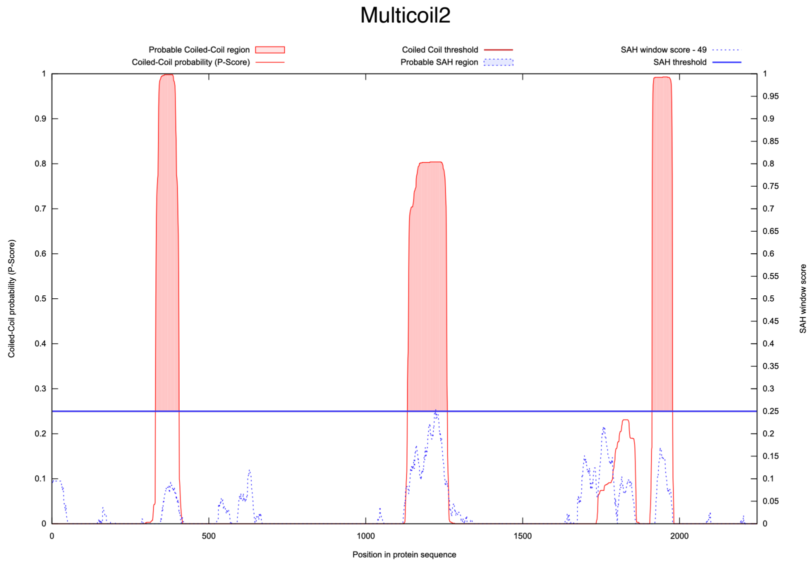
Multicoil2
Probable Coiled-Coil region
Coiled-Coil probability (P-Score)
Coiled Coil threshold
Probable SAH region
SAH window score - 49
SAH threshold
Coiled-Coil probability (P-Score)
SAH window score
Position in protein sequence

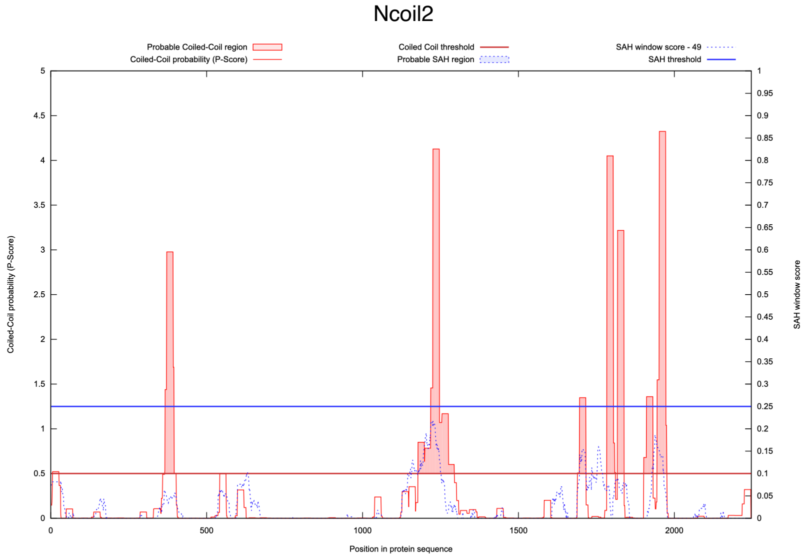
Ncoil2
Probable Coiled-Coil region
Coiled-Coil probability (P-Score)
Coiled Coil threshold
Probable SAH region
SAH window score - 49
SAH threshold
Coiled-Coil probability (P-Score)
SAH window score
Position in protein sequence

Supplementary Figure 8. Prediction of coiled-coil segments within Tme.
Three coiled-coil segments are bioinformatically detected in the Tme polypeptide. The results of the comparative analysis of three different tools are shown. The predictions were performed by Waggawagga[3]. SAH, stable single alpha-helix.

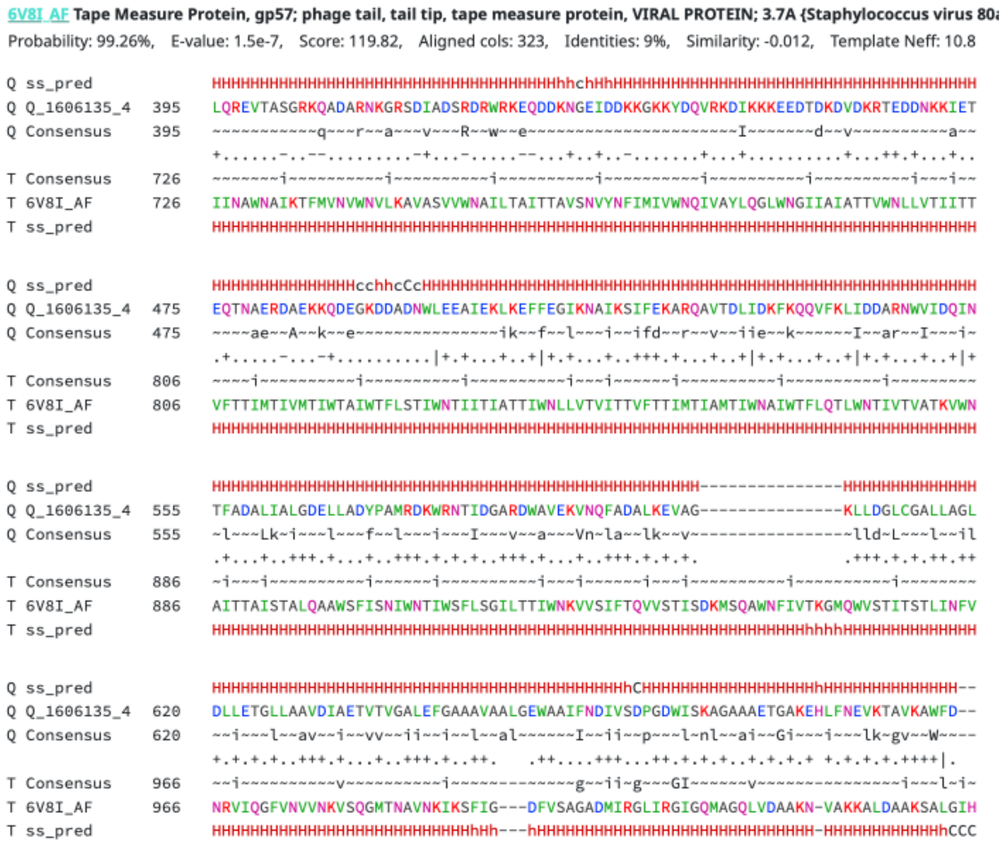

Supplementary Figure 9. Remote homology between a phage tapemeasure protein and Tme.

Homology search for Tme was performed by HHpred using the UniProt database and a representative result is shown. In this figure, a partial amino acid sequence of Tme (query, Q; 1185-1478) was aligned with that of tapemeasure protein of *Staphylococcus aureus* phage 80alpha (template, T; UniProt accession number A4ZFC2_9CAUD; 726-1065). Sequences ss_pred denote the PSI-PRED secondary structure prediction (H, helix; C, coil). Upper and lower case amino acids in the consensus sequences indicate high and moderate conservation, respectively. Symbols indicate the quality of the column-column match: |, very good; +, good; ·, neutral; -, bad.

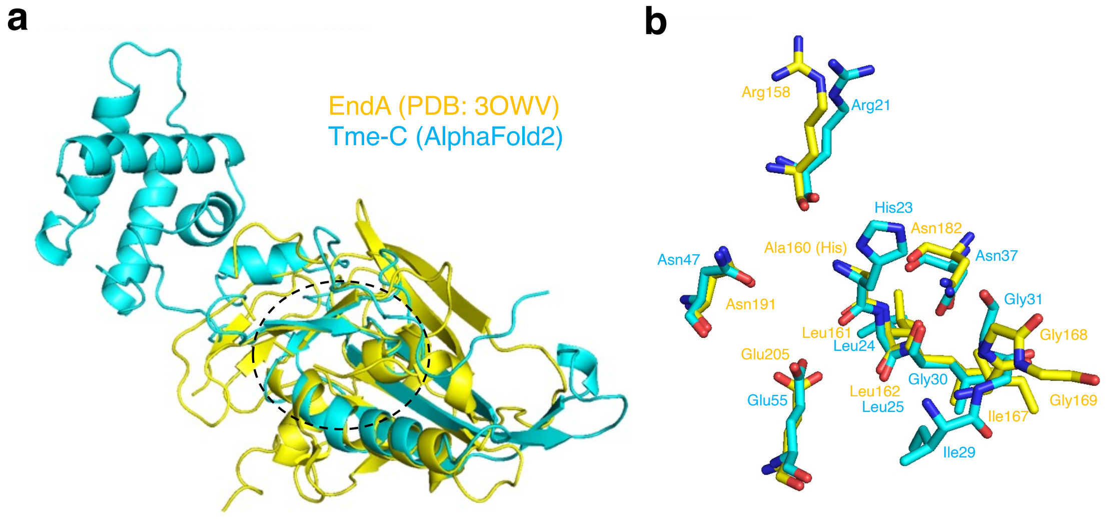

Supplementary Figure 10. The predicted structure of the C-terminal domain of Tme. (a) The AlphaFold2-predicted structure of Tme-C (cyan, the top-ranked model) was superposed with the crystal structure of DNA-entry nuclease EndA of *S. pneumoniae* (yellow, 3OWV). A circle with a dashed line indicates active site of EndA. (b) Amino acid arrangement at the active site is shown. Underlined amino acids of EndA have been proposed to be involved in the nucleolytic reaction. Asn191 and Glu205 of EndA would participate in metal coordination. The probable catalytic histidine His160 of EndA is replaced with Ala in the crystal structure.

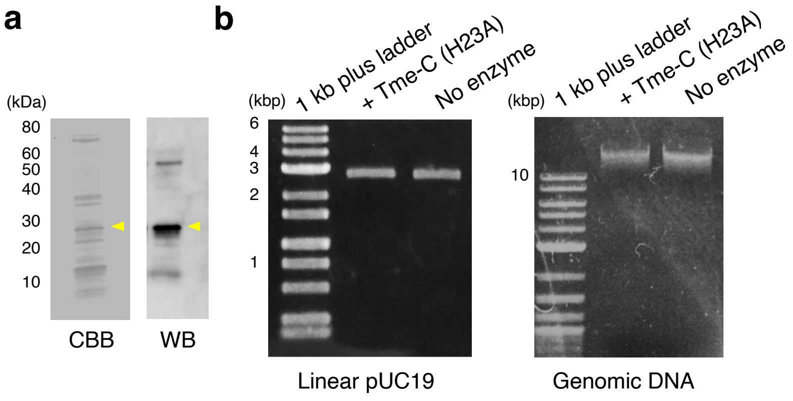

Supplementary Figure 11. Alanine substitution at the predicted catalytic histidine abolished the nucleolytic activity of Tme-C.

(a) Tme-C-His$_6$ (H23A) was heterologously expressed in *E. coli* and the cell lysates were partially purified by Ni^{2+}-affinity chromatography. CBB, Coomassie Brilliant Blue staining. WB, western blotting using anti-Tme-C serum. Yellow arrowheads indicate the bands of Tme-C-His$_6$ (H23A). (b) The partially purified Tme-C (H23A) was reacted with linearised pUC19 (left) and genomic DNA (right) under the same condition with those in Fig. 4f and Supplementary Fig. 7, respectively. All samples were mixed with SDS (0.05% [w/v]) in prior to the electrophoresis. Source data are provided as a Source Data file.

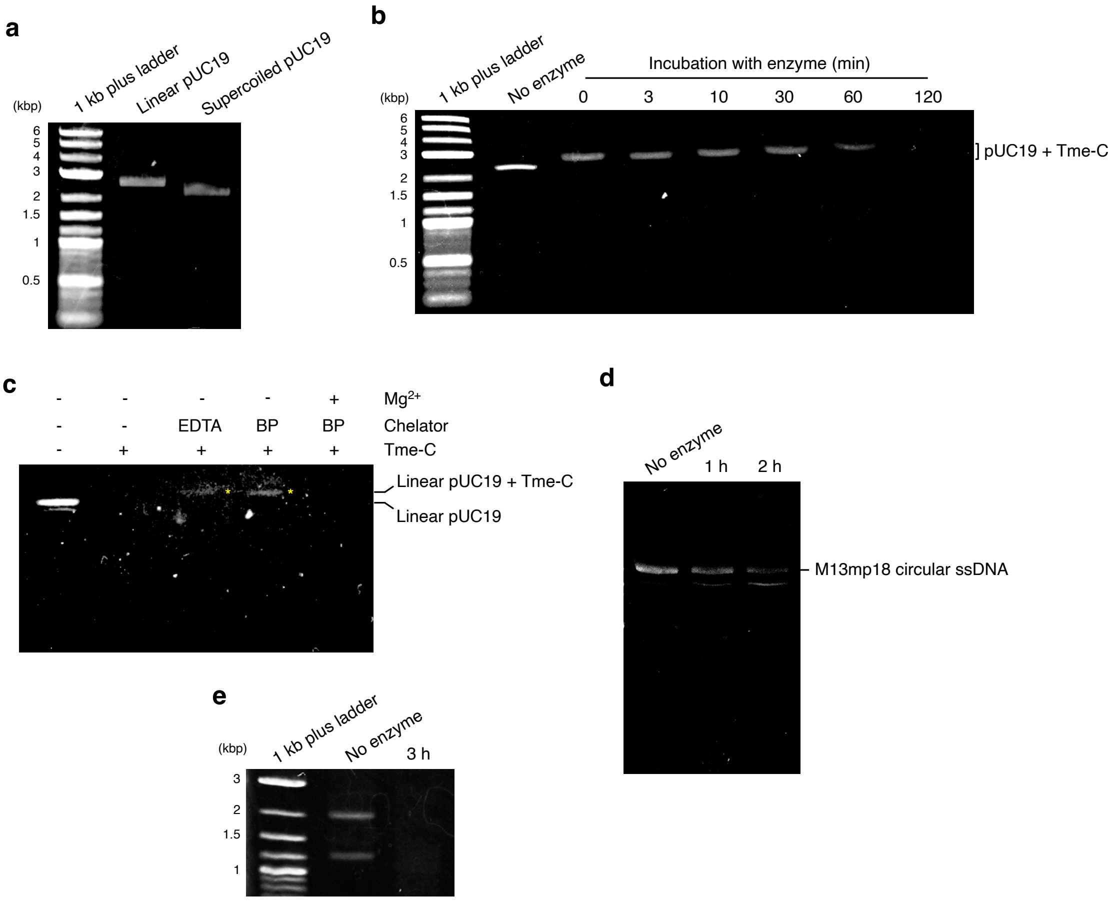

Supplementary Figure 12. Characterisation of Tme-C as an endonuclease.

(a) Double-stranded DNA substrates used in this study are shown. Supercoiled pUC19 migrated faster than its linearised form. (b) Supercoiled pUC19 was reacted with Tme-C. Upward shift of the pUC19 band would indicate the enzyme-substrate complex formed upon the addition of enzyme and break in the supercoiled DNA. (c) Tme-C and linearised pUC19 were reacted for 2 h in the presence of chelators and $MgCl_2$. Yellow stars indicate the bands of the residual substrate appeared upon the addition of weaker (ethylenediaminetetraacetate, EDTA) or stronger (2,2'-bipyridine, BP) chelators. Concentrations of EDTA, BP, and $MgCl_2$ in the reaction mixtures were 10, 2, and 5 mM, respectively. (d) Single-stranded DNA substrate (M13mp18 virion DNA) was reacted with Tme-C. (e) Tme-C was reacted with the extracted RNA. Two bands appearing in the middle lane corresponds to 23S and 16S ribosomal RNA. All samples were mixed with SDS (0.05% [w/v]) in prior to agarose gel electrophoresis.

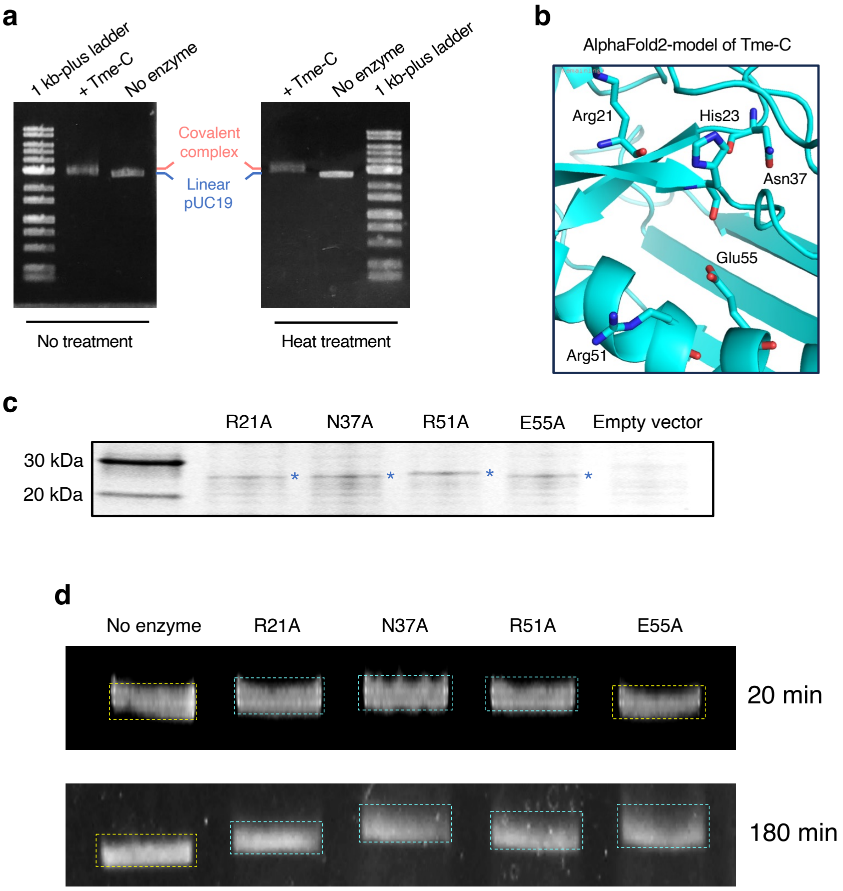

Supplementary Figure 13. Tme-C forms a probable covalent enzyme-substrate complex.

Active site amino acids of Tme-C are involved in the formation of a heat-stable enzyme-substrate complex. (a) Tme-C-His$_6$ (20 ng/µL) was incubated with 5 ng/µL of linearised pUC19 for 20 min and then subjected to agarose gel electrophoresis with or without heat treatment at 95 °C for 3 min. All samples were mixed with SDS (0.05% [w/v]) in prior to the electrophoresis. Upward shift of the band indicates the heat-stable enzyme-substrate complex. (b) The AlphaFold2-model of an active site of Tme-C is shown. His23 is the catalytic amino acids playing a central role in the Tme-C-catalysed reaction. (c) Each of mutant Tme-C-His$_6$ enzymes were heterologously expressed in *E. coli* and partially purified by Ni^{2+}-affinity chromatography. The partially purified proteins were migrated by SDS-PAGE and stained with Coomassie-Brilliant Blue. Asterisks indicate the bands corresponding to the mutant Tme-C-His$_6$ enzymes. (d) Each of the isolated mutant enzyme solutions (20 ng/µL) were incubated with 5 ng/µL of linearised pUC19. All samples were mixed with SDS (0.05% [w/v]) in prior to the agarose gel electrophoresis. Upward shifted and non-shifted bands are indicated by blue and yellow squares, respectively. Source data are provided as a Source Data file.

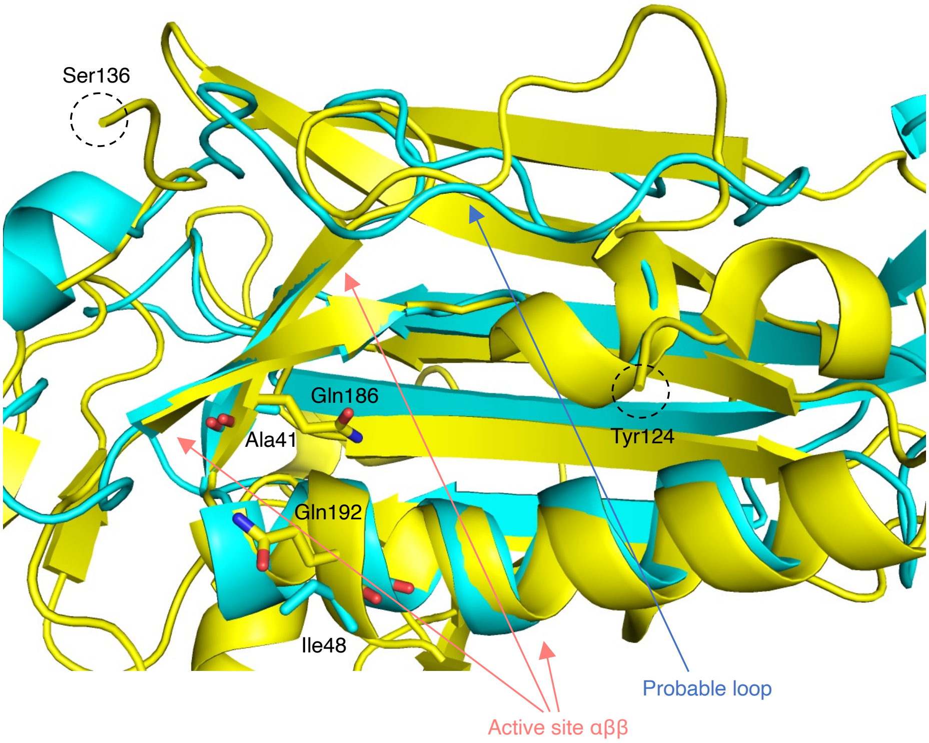

Supplementary Figure 14. Tme lacks several amino acids facilitating substrate binding and DNA hydrolysis.

The AlphaFold2-predicted structure of Tme-C (cyan, the top-ranked model) was superposed with the crystal structure of DNA-entry nuclease EndA of *S. pneumoniae* (yellow, 3OWV). Active site α helix and β strands are indicated by arrows in pale red. Tyr124 and Ser136 of EndA (circles with broken lines) flank the disordered loop containing Arg127/Lys128 that presumably corresponds to “Probable loop” (blue arrow) consisting of the amino acids GGQDPGSAQ in Tme. Gln186 and Gln192 of EndA are replaced with Ala41 and Ile48, respectively, in Tme-C. Details are described under Supplementary Notes.

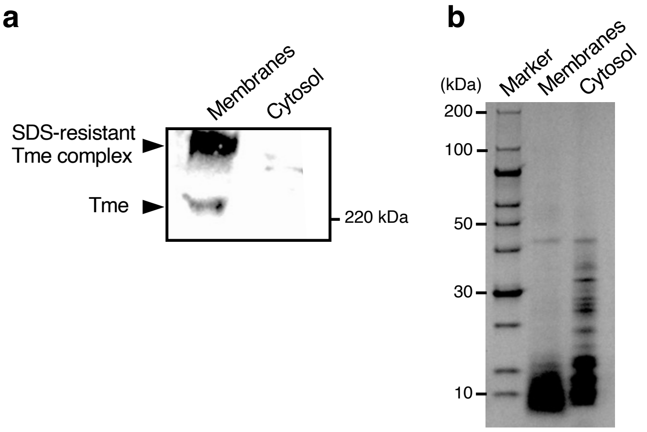

Supplementary Figure 15. Detection of Tme in the isolated membrane fractions. *S. davawensis* mycelia (wildtype) grown for 3 days were disrupted by sonication and then separated into membrane and cytosolic fractions by 0.45 µm filtration and ultracentrifugation/density gradient ultracentrifugation. These fractions isolated from 5 mg (wet weight) of mycelia were subjected to SDS–PAGE and subsequent (a) western blotting using anti-Tme-C serum and (b) CBB staining. Note that the majority of Tme in the membrane sample was detected as an SDS-resistant complex. Source data are provided as a Source Data file.

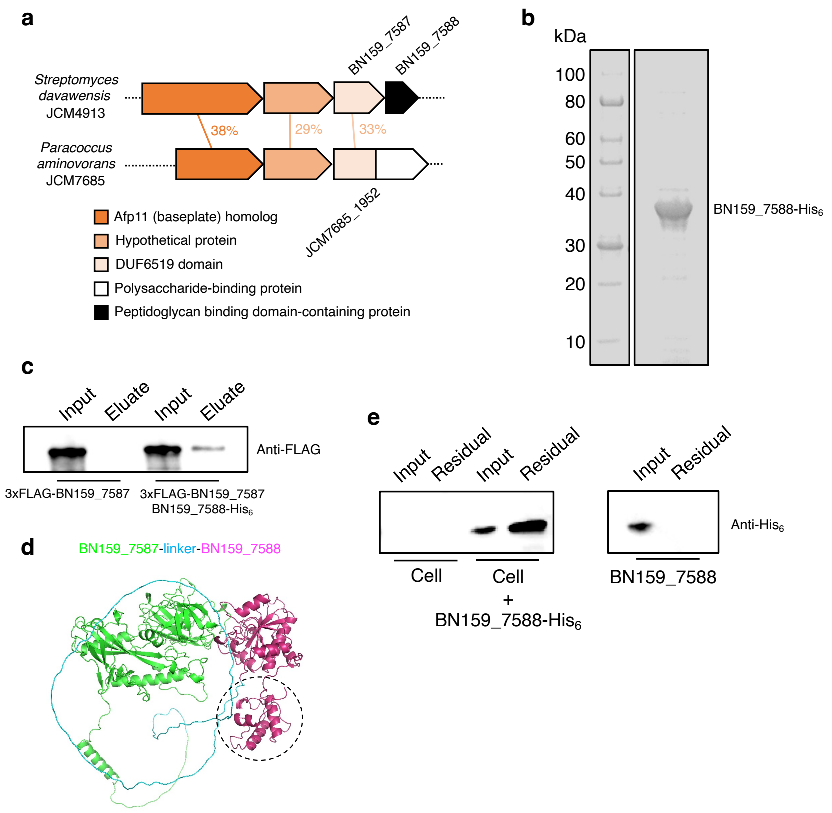

Supplementary Figure 16. A possible role of BN159_7588 in CIS-cell interaction. (a) The genomic context of *BN159_7588* is shown. BN159_7588 is encoded downstream of baseplate-related proteins and is likely to substitute for C-terminal polysaccharide-binding domain of a putative receptor-binding protein-like protein (JCM7685_1952) of the closely related gram-negative bacterial CISs. The percentages indicate amino acid identities. (b) BN159_7588-His_6 was heterologously expressed in *E. coli* and purified by Ni^{2+}-affinity chromatography. (c) 3×FLAG-BN159_7587 was heterologously expressed in *E. coli*, and the lysates were subjected to Ni^{2+}-affinity chromatography with or without BN159_7588-His_6. 3×FLAG-BN159_7587 was detected by western blotting using anti-DDDDK (FLAG) antibody. The band in the eluates indicates the coelution of 3×FLAG-BN159_7587 and BN159_7588-His_6. (d) The AlphaFold2-predicted heterocomplex structure of a putative receptor-binding protein-related protein (BN159_7587, green) and BN159_7588 (pink) is shown. In this structure prediction, a poly-glycine linker (blue) was introduced between these proteins. The top-ranked model is shown. The peptidoglycan-binding domain is indicated by a circle with a broken line. (e) The Ni^{2+}-affinity chromatography-purified protein was

added to the *S. davawensis* mycelia resuspended in phosphate-buffered saline (Input). The mycelia were then washed and isolated by centrifugation (Residual, 10-fold concentrated). The samples without BN159_7587 or mycelia serve as controls. BN159_7588 in each sample was detected by western blotting using anti-His_6 antibody. Source data are provided as a Source Data file.

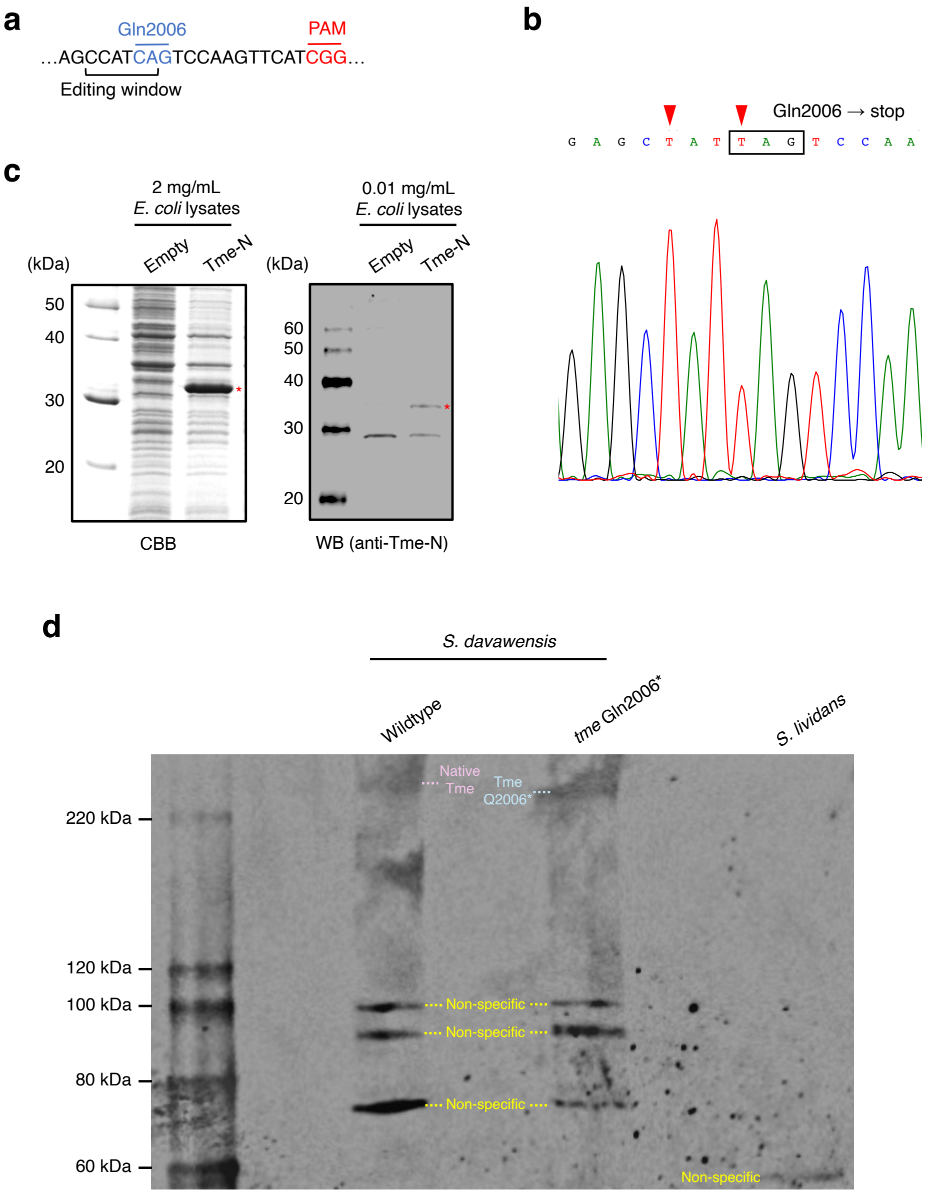

Supplementary Figure 17. Removal of the C-terminal nuclease domain from Tme. (a) Scheme of the Gln2006* mutation in Tme is shown. Cytosine nucleotides within editing window are targeted by APOBEC-Cas9n-UGI enzyme. (b) A representative sequencing result confirming Gln2006* mutation is shown. Red arrowheads indicate the edited nucleotides. (c) Antiserum against the N-terminal region of Tme (Tme-N) was developed as described under the Methods and used for the detection of recombinant Tme-N (amino acids 1-257) expressed in *E. coli*. *E. coli* strains harbouring either pCold::*tme-N* or an empty vector were cultivated and then the cell lysates were subjected to SDS–PAGE. CBB, Coomassie-brilliant blue. WB, western blotting. Red asterisks indicate the bands of Tme-N. (d) Tme (pale red) and its Gln2006* variant (pale blue) were

detected by western blotting using anti-Tme-N serum. The loaded *S. davawensis* lysates of the wildtype strain and the *tme* Gln2006* mutant (10 µg each) are the same as those used in Fig. 4g. The lysates of *S. lividans* mycelia (10 µg) was used as a negative control.

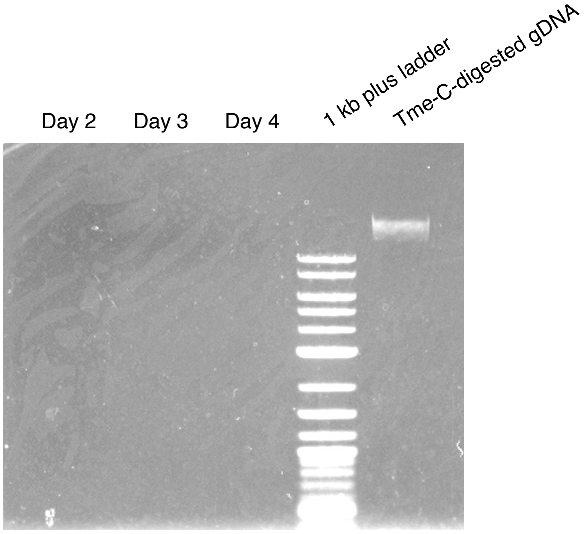

Supplementary Figure 18. High molecular weight eDNA was not detected in the extracted extracellular matrix of the Gln2006* mutant.

The ECM extracts of the Gln2006* mutant were concentrated (10-fold) by ethanol precipitation and migrated by agarose gel electrophoresis. All samples were mixed with SDS (0.05% [w/v]) in prior to the electrophoresis.

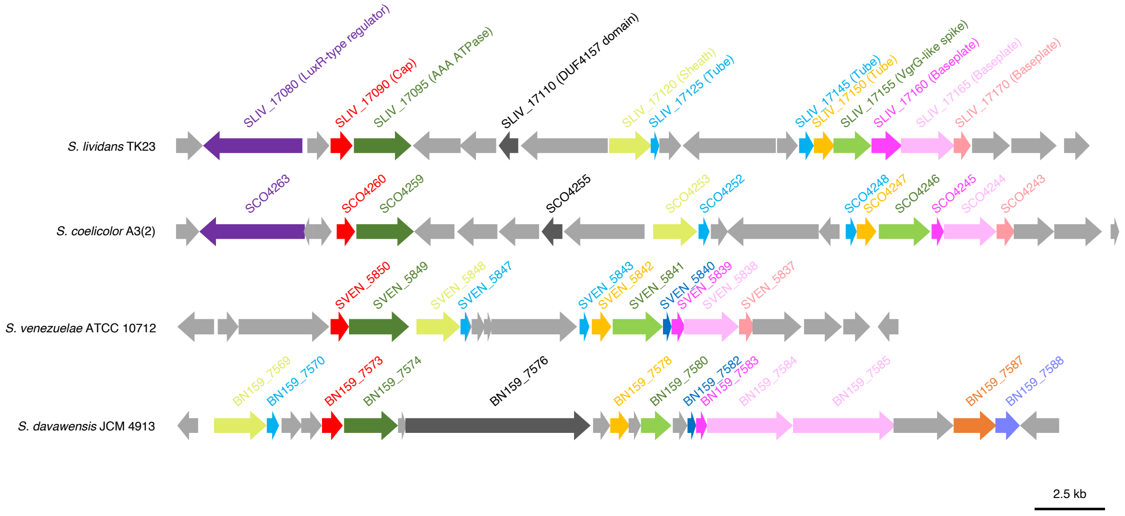

Supplementary Figure 19. Comparison of *Streptomyces* CIS gene clusters.

The previously investigated CISs of the model *Streptomyces* species are compared with the *Streptomyces davawensis* CIS with respect to the synteny and composition of the gene clusters. Gene locus tags corresponds to those registered in GenBank (*S. lividans*, CP009124.1; *S. coelicolor*, AL645882.2; *S. venezuelae*, FR845719.1; *S. davawensis*, HE971709.1). Each colour of arrows indicate the groups of CIS-related proteins. Details are described under Supplementary Notes.

Supplementary Table 1. Bacterial strains used in this study.

Strain	Origin
Streptomyces davawensis JCM4913	RIKEN BRC
Streptomyces davawensis JCM4913 Δ*cisS*	This study
Streptomyces davawensis JCM4913 Δ*cisS*::*cisS*	This study
Streptomyces davawensis JCM4913 pGMHdw:: his_6-*BN159_7582*	This study
Streptomyces davawensis JCM4913 pGMHdw:: *BN159_7582*	This study
Streptomyces davawensis JCM4913 *tme* Gln2006*	This study
Escherichia coli DH5α	Lab stock
Escherichia coli BL21	Lab stock
Streptomyces lividans TK23	Lab stock

Supplementary Table 2. DNA oligomers and plasmids used in this study.

DNA oligomer	Sequence
cBEST_Inverse_Fw	TCTAGAGGATCCCCGGGTACCG
cBEST_Inverse_Rv	GGAACCTATAGTGAGTCGTATT CTGACGCCGTCCACGCTGC
Thy_Riboswitch	AATACGACTCACTATAGGTTCCGGTGATACCAGCATCGTCTTGATGCCCTTGGCAGCACCCTGCTAAGGAGGCAACAAG
cBEST_Overlap_Rv	CGGAGGACATATG CTTGTTGCCTCCTTAGCAGGGTGC
CisS_pTYM19t_Fw	ATGATTACGCCAAGCTT GAGGGCATGCGTGTTCGAGTC
CisS_pTYM19t_Rv	AGTGAATTCGAGCTCGGTACC CGGTTCCCTCGTGGATCTCGT
Tme_Fw	AGAAGGAGATATACAT ATGAGCAACTCCCGTGCGCAGG
Tme_Rv	AGTGCGGCCGCAAGCTT TCAACTCCAGCTCACTTCGCCGGC
H2051A_Fw	GCA CTGCTGCCCGAGCGGATCGG
H2051A_Rv	CATGCGGACCCAGCGGGCG
Tme-C_Fw	GAAGGAGATATACAT ATGAATCACAAAGTG CCACCAGGTTGGGGCGATGT
Tme-C_Rv	AGTGCGGCCGCAAGCTT GGACCACGACACTTCTCCCGC
H23A_Fw	CTGCTTCCCGAACGCATTGGTG
H23A_Rv	TGC CATGCGTACCCAACGCGCAC
pGMH_Inverse_Fw	CAACGTTCCGAGAGGTTGTTC
pGMH_Inverse_Rv	GTCATAGCTGTTTCCTGTGTGAAATTGT
PhrdB_Fw	GGAAACAGCTATGAC GCCCCGCGCGAGCACTGAC
PhrdB_Rv	GAACAACCTCTCGGAACGTTGGAAAACGGC
Tip_Fw	TCCGAGAGGTTGTTC A TGTCCGGGAGTCTGCTCGACGC
Tip_Rv	CCGGGGATCTAAGCT TCATCGGACGGTGACCTTTCGCTG
His6-GGGSx2-Tip_Fw	TCCGAGAGGTTGTTC ATG CACCACCACCACCACCAC GGCGGTGGCGGGTCCGGCGGTGGCGGGTCC A TGTCCGGGAGTCTGCTCGACGC
CisS_Q78_1	GGTAGGATCGACGGC GTCCACCAGTTCTTCGGCAA GTTTTAGAGCTAGAA
CisS_Q78_2	TTCTAGCTCTAAAACTTGCCGAAGAACTGGTGGACGCCGTCGATCCTACC
Tme_Q2006_1	GGTAGGATCGACGGC AGCCATCAGTCCAAGTTCAT GTTTTAGAGCTAGAA
Tme_Q2006_2	TTCTAGCTCTAAAACATGAACTTGGACTGATGGCTGCCGTCGATCCTACC
BN159_7588_Fw	AGAAGGAGATATACAT ATGGCCAAGCCGCTGAGCGC
BN159_7588_Rv	AGTGCGGCCGCAAGCTT GTTGGGCCCGTTCGGCACCTTC
BN159_7588_Fw	ATAAGGGCGGTGGCGGTAGCATGCACGCTGACCTCTCCCGC
BN159_7588_Rv	GGTGCTCGAGTGCGGCCGCATCACTCGTCTCCTTCCGCCGTG
3xFLAG	CTTTAAGAAGGAGATATACATATGGACTACAAGGACCACGACGGCGACTACAAGGACCACGACATCGATTACAAGGACGATGACGATAAGGGCGGTGGCGGTAGC
R21A_Fw	ATGCATCTGCTTCCCGAACGC
R21A_Rv	TGCTACCCAACGCGCACCTTCGC
N37A_Fw	CTGGTTCCTGCACGTGGTCCG
N37A_Rv	TGCGTTGCCAGTGGCTCTACCACCA
R51A_Fw	GAAGAGATTGAAGACAAAGCGTATCAGG
R51A_Rv	TGCGGCTTTGATGTTGGTTTCCGGAC
E55A_Fw	GACAAAGCGTATCAGGCGATTGGC
E55A_Rv	TGCAATCTCTTCGCGGGCTTTGATGTTG
Tme-N_Fw	TACCCTCGAGGGATCCATGAGCAACTCCCGTGCGCA
Tme-N_Rv	TGCAGGTCGACAAGCTTTCACTGCTCGGTCTCCTCGACCAG

Plasmid	Description
pCRISPR-cBEST-RS	Theophylline-dependent genome editing system
pCRISPR-cBEST-RS_Q78	Theophylline-dependent genome editing system for *cisS*
pCRISPR-cBEST-RS_Q2006	Theophylline-dependent genome editing system for *tme*
pTYM19t::*cisS*	Complementation of *cisS*
pGMHdw:: *his_6-BN159_7582*	Expression of His_6-Tip
pGMHdw:: *BN159_7582*	Expression of Tip
pET26b::*tme*	Expression of Tme
pET26b::*tme* (H2051A)	Expression of Tme with H2051A mutation
pET26b::*tme-C-his_6*	Expression of Tme-C
pET26b::*tme-C* (H23A)-*his_6*	Expression of Tme-C with H23A mutation
pET26b::*tme-C* (R21A)-*his_6*	Expression of Tme-C with R21A mutation
pET26b::*tme-C* (N37A)-*his_6*	Expression of Tme-C with N37A mutation
pET26b::*tme-C* (R51A)-*his_6*	Expression of Tme-C with R51A mutation
pET26b::*tme-C* (E55A)-*his_6*	Expression of Tme-C with E55A mutation
pET26b::*BN159_7588-his_6*	Expression of BN159_7588
pET26b::*3xFLAG-BN159_7587*	Expression of BN159_7587
pCold::*tme-N*	Expression of Tme-N

Supplementary Table 3. A list of CIS tube proteins.

Origin	Accession	Gene locus tag
Enterobacteria phage T4	NP049781	T4p168
Serratia entomophila plasmid pADAP	AF135182	AAT48338.1
Photorhabdus asymbiotica ATCC 43949	FM162591	PAU_03353
Algoriphagus sp. PR1	CM001023	ALPR12750
Desulfobacca acetoxidants DSM 11109	CP002629	Desac_1803
Pelobacter propionicus DSM 2379	CP000482	Ppro_1826
Lysobacter enzymogenes M497-1	AP014940	LEN_1488
Micromonospora echinofusca DSM 43913	LT607733	GA0070610_2943
Ralstonia solanacearum KACC 10722	CP14702	LBM2029_08945
Myxococcus stipitatus DSM 14675	CP004025	MYSTI_06891
Mycobacterium sp. JS623 plasmid pMYCSM01	CP003079	Mycs06568
Bradyrhizobium sp. ORS278	CU234118	BRA004389
Candidatus Koribacter versatilis Ellin345	CP000360	Acid30862
Streptomyces avermitilis MA-4680	BA000030	SAVERM_382
Streptomyces davawensis JCM 4913	HE971709	BN159_7570
Lysobacter antibioticus strain 76	CP011129	LA76x_2811
Paracoccus aminovorans JCM 7685	LN832559	JCM7685_1936
Chloroflexus aggregans DSM 9485	CP001337	Cagg_1504
Streptomyces coelicolor A3(2)	AL645882	SCO4252
Streptomyces lividans TK24	CP009124	SLIV17125

Supplementary Table 4. A list of CIS sheath proteins.

Origin	Accession	Gene locus tag
Enterobacteria phage T4	NP049781	T4p167
Serratia entomophila plasmid pADAP	AF135182	AAT48341.1
Photorhabdus asymbiotica ATCC 43949	FM162591	PAU_03350
Algoriphagus sp. PR1	CM001023	ALPR12755
Desulfobacca acetoxidants DSM 11109	CP002629	Desac_1802
Pelobacter propionicus DSM 2379	CP000482	Ppro_1827
Lysobacter enzymogenes M497-1	AP014940	LEN_1487
Micromonospora echinofusca DSM 43913	LT607733	GA0070610_2942
Ralstonia solanacearum KACC 10722	CP14702	LBM2029_08950
Myxococcus stipitatus DSM 14675	CP004025	MYSTI_06890
Mycobacterium sp. JS623 plasmid pMYCSM01	CP003079	Mycs06569
Bradyrhizobium sp. ORS278	CU234118	BRA004390
Candidatus Koribacter versatilis Ellin345	CP000360	Acid30863
Streptomyces avermitilis MA-4680	BA000030	SAVERM_381
Streptomyces davawensis JCM 4913	HE971709	BN159_7569
Lysobacter antibioticus strain 76	CP011129	LA76x_2810
Paracoccus aminovorans JCM 7685	LN832559	JCM7685_1935
Chloroflexus aggregans DSM 9485	CP001337	Cagg_1505
Streptomyces coelicolor A3(2)	AL645882	SCO4253
Streptomyces lividans TK24	CP009124	SLIV17120

Supplementary Notes

1. Tme as an effector protein non-lethal in S. davawensis

We consider that the Tme activity would not be physiologically detrimental to *S. davawensis* for the following reasons.
(*i*) Endonuclease activity of Tme would be significantly low compared with other EndA-related endonucleases (see also the section *4. Unique enzymatic characteristics of Tme*). Probably due to the low activity, the isolated Tme-C (endonuclease-like C-terminal domain of Tme) could degrade genomic DNA only partially (Supplementary Fig. 7). Notably, *Streptomyces* mycelia are multinucleated[4], potentially allowing diffusible transcription/translation machineries to access multicopy of genomic DNA during the developmental cycle. On the other hand, access of Tme to genomic DNA would be physically restricted by its association to the cellular membrane (Supplementary Fig. 15), suggesting that only genomic DNA in the proximity of the cellular membrane is targeted by Tme. This could leave the majority of genomic DNA in cytosolic space intact. Moreover, even if all copies of genomic DNA within a single mycelium were targeted by Tme, its low activity would limit DNA degradation to a partial level and allow continued expression of essential genes, further ensuring cellular survival. Additionally, since the Tme polypeptide containing probable disordered regions is predicted to be unstable in solution (instability index (II) is 46.01, above instability threshold 40), it can be speculated that, in the absence of a CIS particle that can load Tme inside its lumen, Tme might be folded improperly in an aqueous solution and/or might form an inactive complex with itself and/or other proteins[5]. The structural instability of Tme in solution may prevent delocalised Tme from fully exhibiting its activity, further ensuring that the producer bacterium *S. davawensis* circumvents the potentially detrimental consequence of the nucleolytic activity of Tme.

(*ii*) CIS particle would also safeguard *S. davawensis* by preventing Tme from accessing genomic DNA uncontrollably. Tme is encoded downstream of the sheath/tube and upstream of baseplate complex proteins (Fig. 1a). Tandem arrangement of CIS proteins with overlapping open reading frames or short intergenic regions ($<$40 bases) may allow them to interact immediately after translation from possible polycistronic mRNA. Following baseplate complex assembly, Tme will be loaded into the CIS particle,

ensuring its controlled release.

2. Comparison of Streptomyces CIS gene clusters

There are several critical differences between *S. davawensis* CIS and the previously investigated *Streptomyces* CISs with respect to the synteny and composition of the gene clusters (Supplementary Fig. 19). Among them, only the *S. davawensis* CIS gene cluster encodes a singular Afp1/5 homolog (BN159_7570; tube protein) and two Afp11 homologs (BN159_7584 and BN159_7585; baseplate proteins). In addition, hypothetical proteins are encoded downstream of each of BN159_7578 (Afp7 homolog; tube initiator protein) and BN159_7580 (Afp8 homolog; VgrG-like spike protein). Although these hypothetical proteins are annotated as Afp7/8 homologs in a previous study[6] (http://www.mgc.ac.cn/cgi-bin/dbeCIS/showecis.cgi?id=GCA_000349325.1), in the current study, we could not confirm their significance as CIS structural proteins since these proteins were not detected in the isolated BN159_7578-BN159_7580 spike complex (Fig. 3). Still, the potential multiplicity of the spike proteins is likely to be a general feature of the CIS gene clusters harbouring Tme-like genes (Supplementary Fig. 2). Probable tail fiber proteins (BN159_7587 and BN159_7588) are present only in the *S. davawensis* CIS, suggesting that the mechanism of target recognition would be different between the *S. davawensis* CIS and the other *Streptomyces* CISs. Finally, DUF4157 domain, a proposed core domain of CIS effectors, can be found in SLIV_17110 and SCO4255 besides BN159_7576 (Tme). Although this imply the potential relevance of SLIV_17110 and SCO4255 to the CISs, their function(s) should be quite different from that of Tme because these DUF4157 domain-containing proteins lack the following key structural features of Tme: the predicted coiled-coil/disordered regions homologous to phage tapemeasure proteins, the central hydrophobic segments predicted to form transmembrane helices, and the C-terminal enzymatic domain. Collectively, these observations suggest that the *S. davawensis* CIS would be functionally and structurally distinct from the previously investigated *Streptomyces* CISs.

3. Biological significance of CISs among Streptomyces species

Genome-wide analyses have shown that gene clusters encoding sets of CIS-related proteins are conserved in ~90% of *Streptomyces* species and most of these CIS proteins

belong to an actinomycetes-exclusive CIS lineage[6]. While these observations suggest that these "typical" actinomycetes CISs have propagated vertically during divergence of *Streptomyces* species from a common ancestor, previous studies have consistently demonstrated the non-essentiality of these CISs for the morphological differentiation in at least two model species *S. lividans* and *S. coelicolor*[7,8,9]. Rather, this type of CISs is likely to confer ecological benefits to the producer bacteria by indirectly affecting the differentiation under certain ecological conditions[7,8,9]. This would imply that, despite their high conservation among *Streptomyces*, CISs have been employed by these bacteria as non-essential systems which indirectly contribute to their life cycle. Importantly, *Streptomyces* species typically possess only a single CIS gene cluster within a genome, possibly due to potential promiscuity in subunit-subunit interactions among different types of CISs resulting in misassembly of protein complexes. Therefore, the non-essentiality of "typical" actinomycetes CIS would allow for the replacement with the "unique" CIS, such as the Tme-harbouring CIS analysed in the current study, in a *Streptomyces* species when both CISs affect the morphological differentiation, and the latter CIS is more advantageous to the bacterium. Ecological niches each *Streptomyces* species has established, and the niche-associated selective pressures would determine which types of CISs are more advantageous and ultimately drive the selection of CISs.

4. Unique enzymatic characteristics of Tme-C

We believe that low nucleolytic activity of Tme-C allowed some of the *E. coli* cells to grow slowly and continue to express this enzymatic domain during cultivation. In our experiments, digestion of 2.5 ng/μL of super-coiled pUC19 plasmid (2.69 kbp) by 50 ng/μL of Tme-C (~25 kDa) was observed after 60-120 min of incubation (Supplementary Fig. 12). This suggests significantly lower nucleolytic activity compared to reactivated EndA H160G mutant (~30 kDa), which could digest 15 ng/μL of super-coiled pBluescript SK(+) plasmid (2.96 kbp) in 30-35 min at ~0.6 ng/μL, while differences in the reaction conditions exist[10]. There are several data further implying the low nucleolytic activity of Tme-C and Tme.

(*i*) Two amino acids contributing to substrate binding in *S. pneumoniae* EndA are not conserved in Tme. Arg127/Lys128 of EndA are located at a disordered loop between Tyr124 and Ser136 connecting active site β-strands (Supplementary Fig. 14). They have been proposed to interact with negatively charged phosphate moiety of substrate nucleotides, contributing to substrate binding[10]. On the other hand, the amino acid

sequence presumably corresponding to the loop is GGQDPGSAQ in Tme, lacking the positively charged Arg/Lys pair. This suggests that the initial interaction between Tme and the phosphate moiety may be weaker compared to EndA. Consequently, the orientation of substrate nucleotides approaching the catalytic site could be more variable, potentially leading to a less efficient Tme-catalysed nucleolytic reaction.

(*ii*) Two amino acid residues facilitating the nucleolytic reaction would be nonreactive in Tme. Gln186 and Gln192 located at an active site β-sheet and α-helix, respectively, of EndA appear to be substituted with less reactive Ala41 and Ile48, respectively, in the predicted Tme-C structure (Supplementary Fig. 14). Given that the alanine-mutagenesis on each of these amino acids markedly decreased the hydrolytic activity but not substrate binding in EndA, it seems that the system facilitating hydrolysis of DNA substrate is not fully functional in Tme-C[10]. Nevertheless, Tme-C can act as an endonuclease since the essential amino acids (His23 and the probable metal-coordinating amino acids) directly involved in the catalysis are conserved (Supplementary Fig. 10).

(*iii*) Tme-C is likely to form a probable covalent enzyme-substrate complex that potentially decreases catalytic turnover rate. We have consistently observed that the band of a double-stranded DNA substrate in an agarose gel shifts upward upon the addition of Tme-C and then gradually disappears (Supplementary Fig. 12b). Importantly, the shifted bands remained after the addition of SDS (0.05% (w/v) at a final concentration) and heat treatment (3 min at 95 °C), suggesting the formation of a probable covalent Tme-C-substrate complex (Supplementary Fig. 13a). The formation of a probable covalent complex might be a unique reaction step of Tme-C as the proposed reaction mechanism of EndA is initiated by activation of a water molecule by H160 and would not undergo the formation of a covalent intermediate[10]. A possible mechanism of the complex formation is nucleophilic addition of an active site amino acid residue to phosphorus of DNA substrates. In PLD superfamily endonucleases including Nuc, a phosphohistidine intermediate containing heat-labile P-N bond is formed *via* nucleophilic addition of an active site His to phosphorus of DNA substrates[11]. However, in the case of Tme-C, the formation of the P-N bond-containing phosphohistidine can be ruled out due to the heat-stable property of the complex (Supplementary Fig. 13a). Therefore, we consider that nucleophilic addition of a R-OH residue (where R can be CH_2, C(=O), or benzene) on the phosphorus, generating rather stable P-O-C bond, is more likely pathway. Since the formation of a probable covalent complex is dependent on the catalytic His23, imidazole residue of His23 possibly interacts with the phosphate moiety or activates the putative phosphate acceptor, facilitating the complex formation. To gain more insight into the complex formation, we conducted an alanine substitution mutagenesis for the predicted

active site amino acids of Tme-C (Supplementary Fig. 13b-d). We targeted Arg21, Asn37, Arg51, and Glu55 that were predicted to surround the catalytic His23 of Tme-C (Supplementary Fig. 13b). Arg21Ala, Asn37Ala, and Arg51Ala mutant enzymes seemed to be reactive with respect to the complex formation. On the other hand, Glu55Ala mutation seemed to inhibit the complex formation with 20 min of incubation (Supplementary Fig. 13d, top). Given that the Glu55 counterpart Glu205, as well as the catalytic His160, has been found as the most crucial amino acid for the EndA-catalysed reaction[10], it is reasonable to consider that the formation of a probable covalent Tme-C-substrate complex would involve the active site residues. Although the detailed role of Glu55 in the complex formation remains to be determined, Glu55 is unlikely to directly accept the phosphate moiety since a probable covalent complex was eventually formed after the prolonged incubation with the substrate (Supplementary Fig. 13d, bottom). Rather, Glu55 may coordinate DNA substrates *via* interaction with water molecule(s) or magnesium ion[10], thereby facilitating the His23-mediated complex formation. His23 and Glu55 would then catalyse hydrolysis of P-O-C bond similarly to EndA by activating a water molecule to attack on phosphorus. The formation of the relatively stable complex and multiple roles of the active site residues could significantly limit the nucleolytic activity of Tme-C compared with EndA-related endonucleases in which the catalytic residues would be dedicated to hydrolysing the DNA phosphodiester. Interestingly, a probable covalent complex was not detected when single-stranded DNA was used as a substrate, suggesting that the conformation of substrate nucleotides approaching the active site may critically affect the efficiency of an enzyme-substrate interaction and consequently the complex formation.

A covalent enzyme-DNA intermediate containing a transient P-O-C bond is formed during the type I topoisomerase-catalysed cleavage of the DNA phosphodiester, followed by religation of DNA[12]. Notably, type I topoisomerase has been shown to be evolutionarily related to endonucleases[13] and be converted into an endonuclease through the replacement of a phosphate acceptor Tyr containing hydroxyl group with other amino acids such as Glu[14]. These observations suggest a mechanistic similarity of endonuclease reactions and the type I topoisomerase-catalysed DNA cleavage, the latter involving the formation of the stable, covalent DNA-enzyme complex. It is thus speculated that some enzymes catalysing endo-hydrolysis of the DNA phosphodiester might have the potential to form transient, covalent enzyme-DNA complexes depending on the presence of a proper phosphate-acceptor residue. Although this hypothesis requires further investigation before applying it to the Tme-C-catalysed reaction, solving a high-resolution structure of the Tme-C-substrate complex will help identify a putative

phosphate acceptor residue and reveal the detailed mechanism of the unique Tme-C-catalysed reaction.

Supplementary References

1. Gilchrist, C. L. M. and Chooi, Y. H. clinker & clustermap.js: Automatic generation of gene cluster comparison figures. *Bioinformatics* **37**, 2473-2475 (2021).

2. Krogh, A., Larsson, B., von Heijne, G., Sonnhammer, E. L. L. Predicting transmembrane protein topology with a hidden Markov model: Application to complete genomes. *J. Mol. Biol.* **305**, 567-580 (2001).

3. Simm, D., Hatje, K., Kollmar, M. Waggawagga: comparative visualization of coiled-coil predictions and detection of stable single α-helices (SAH domains). *Bioinformatics* **31**, 767-769 (2015).

4. Yaqüe, P., López-García, M. T., Rioseras, B., Sánchez, J., Manteca, Á. Pre-sporulation stages of *Streptomyces* differentiation: state-of-the-art and future perspectives. *FEMS Microbiol. Lett.* **342**, 79-88 (2013).

5. van der Lee, R. *et al.* Classification of intrinsically disordered regions and proteins. *Chem. Rev.* **114**, 6589-6631 (2014).

6. Chen, L., Song, N., Liu, B., Zhang, N., Alikhan, N. F., Zhou, Z., Zhou, Y., Zhou, S., Zheng, D., Chen, M., Hapeshi, A., Healey, J., Waterfield, N. R., Yang, J., Yang, G. Genome-wide identification and characterization of a superfamily of bacterial extracellular contractile injection systems. *Cell Rep.* **29**, 511–521 (2019).

7. Casu, B., Sallmen, J. W., Schlimpert, S., Pilhofer, M. Cytoplasmic contractile injection systems mediate cell death in *Streptomyces*. *Nat. Microbiol.* **8**, 711-726 (2023).

8. Vladimirov, M., Zhang, R. X., Mak, S., Nodwell, J. R., Davidson, A. R. A contractile injection system is required for developmentally regulated cell death in *Streptomyces coelicolor*. *Nat. Commun.* 14:1469 (2023).

9. Nagakubo, T., Yamamoto, T., Asamizu, S., Toyofuku, M., Nomura, N., Onaka, H.

Phage tail-like nanostructures affect microbial interactions between *Streptomyces* and fungi. *Sci. Rep.* 11:20116 (2021).

10. Moon, A. F., Midon, M., Meiss, G., Pingoud, A., London, R. E., Pedersen, L. C. Structural insights into catalytic and substrate binding mechanisms of the strategic EndA nuclease from *Streptococcus pneumoniae*. *Nucleic Acid Res.* **39**, 2943-2953 (2011).

11. Gottlin, E. B., Rudolph, A. E., Zhao, Y., Matthews, H. R., Dixon, J. E. Catalytic mechanism of the phospholipase D superfamily proceeds via a covalent phosphohistidine intermediate. *Proc. Natl. Acad. Sci. USA* **95**, 9202-9207 (1998).

12. Znag, Z., Cheng, B., Tse-Dinh, Y. C. Crystal structure of a covalent intermediate in DNA cleavage and rejoining by *Escherichia coli* DNA topoisomerase I. *Proc. Natl. Acad. Sci. USA* **108**, 6939-6944 (2011).

13. Huai, Q., Colandene, J. D., Chen, Y., Luo, F., Zhao, Y., Topal, M., Ke, H. Crystal structure of *Nae*I—an evolutionary bridge between DNA endonuclease and topoisomerase. *EMBO J.* **19**, 3110-3118 (2000).

14. Wittschieben, J., Petersen, B., Shuman, S. Replacement of the active site tyrosine of vaccinia DNA topoisomerase by glutamate, cysteine or histidine converts the enzyme into a site-specific endonuclease. *Nucleic Acids Res.* **26**, 490-496 (1998).
